# Peer navigation for individuals with serious mental illness leaving jail: a pilot randomized trial study protocol

**DOI:** 10.1186/s40814-020-00659-1

**Published:** 2020-08-17

**Authors:** Maji Hailemariam, Lauren M. Weinstock, Jennifer E. Johnson

**Affiliations:** 1 Department of Obstetrics, Gynecology and Reproductive Biology, College of Human Medicine, Michigan State University, East Lansing, MI USA; 2 Department of Psychiatry & Human Behavior, Alpert Medical School of Brown University, Providence, RI USA; 3 College of Human Medicine, Division of Public Health, Michigan State University, Flint, MI USA

## Abstract

**Background:**

Serious mental illness (SMI) is a prevalent public health problem affecting 25% of individuals in jail. Re-entry to the community following incarceration is a vulnerable time for justice-involved individuals with SMI. SMI requires prompt and ongoing access to mental health and other healthcare services.

**Methods:**

The study will (1) develop a Mentoring And Peer Support (MAPS) intervention for post-release mental health and other service connection among jailed individuals with SMI and (2) pilot test the MAPS intervention to determine its feasibility and acceptability. The primary outcomes will be to evaluate the feasibility and acceptability of the proposed recruitment methods and research design, of the intervention training methods, and of delivering the enhanced peer-navigator and control interventions. Study samples include focus groups (n=36), open trial (n=15), and a randomized pilot trial in a sample of 40 individuals with SMI re-entering the community after jail release. Secondary outcomes will include post-release enrollment in mental health, medical care, and substance use services. We will also evaluate reduction in psychiatric symptoms, improvements in functioning, adherence to psychiatric medications, fewer substance using days, fewer hospitalizations and suicide attempts, nights unstably housed, and time until rearrest.

**Discussion:**

This pilot study will evaluate the feasibility and acceptability of a peer navigation intervention for individuals with serious mental illness leaving jails. The study will serve as a formative work for a larger randomized controlled trial assessing the effectiveness of peer navigator intervention for (include the primary outcome) in this population.

## Background

The US criminal justice system contributes to 22% of the world’s incarcerated population [1]. Approximately 12 million people in the US become or are incarcerated each year. Among these, individuals with serious mental illnesses (SMI; psychotic and affective disorders associated with functional impairment and interference with major life activities) are disproportionately represented, accounting for 25% of the justice-involved population [2–4].

Re-entry to the community following incarceration is a vulnerable time for justice-involved individuals with SMI. They experience multiple barriers to accessing mental health, medical care (preventive and curative), and social services due to debilitating symptoms, practical challenges accessing community services, and the stigma associated with being diagnosed with SMI [5, 6]. Potential consequences of lack of care include exacerbation of psychiatric symptoms, substance use relapse, homelessness, and repeat incarceration [6–8].

Peer navigation has been found to improve access to mental health services and medical care among non-justice involved individuals [9, 10]. Peer navigators are individuals who have successfully overcome an adverse life experience and whose lived experiences and formal trainings [11] are believed to support or encourage others in similar situations [12]. Peer navigators share life experiences with their clients such as a diagnosis of SMI or a history of CJ involvement, making them credible and persuasive as navigators than non-peer navigators [13]. Peer-provided services are positively regarded and accepted and are often preferred by individuals with SMI because they demonstrate better understanding compared to regular case managers [14]. Three previous studies of non-justice involved individuals with SMI found that SMI peer navigators increase adherence to psychotropic medications [15] and medical and mental health care utilization [16, 17].

No peer support interventions have been tested to assist with mental health service linkage for individuals with SMI during re-entry to the community after jail incarceration. One study of substance use peer navigators during community re-entry (not among individuals with SMI) has shown that using a peer navigator model during re-entry is feasible [5]. The purpose of the proposed study is to develop a peer navigator intervention for mental health, substance use, and medical care treatment engagement among justice-involved individuals with SMI who are re-entering the community after jail incarceration and to then evaluate its feasibility, acceptability, and ways the intervention contributed to the desired outcomes.

In this study, peer navigators will be trained individuals who share the joint experience of having the diagnosis of SMI and also having a history of justice involvement. Their lived experience as someone diagnosed with SMI and familiarity with the justice system makes them ideal practical supports for facilitating prompt and ongoing access to mental health, medical care, and substance use services for others at community re-entry.

This pilot study will provide formative work for a larger randomized controlled trial evaluating the effectiveness of peer navigator intervention for justice-involved individuals with SMI. The study will have two phases. The first (development) phase aims to (1) enhance and tailor a peer-navigator intervention for justice-involved individuals with SMI re-entering the community using qualitative research; (2) develop, implement, and evaluate the intervention training program using qualitative and quantitative techniques; and (3) improve the clarity, content, acceptability, and feasibility of the tailored peer navigator intervention through a small open trial (*n* = 15) of justice-involved individuals with SMI re-entering the community. The second (pilot study) phase aims to conduct a randomized pilot trial in a sample of 40 justice-involved individuals with SMI who are anticipating jail release in the next 30 days. The control condition will be Standard of Care (SOC). Study assessments will take place at baseline, 3 months, and 6 months after release. The goal is to demonstrate the feasibility and acceptability of the proposed recruitment methods and research design, of the intervention training methods, and of delivering the enhanced peer-navigator and control interventions.

This study protocol describes the planned process of development of a peer navigation intervention for creating linkages to mental health, medical care, and substance use services. We also present plans for evaluation of feasibility, acceptability, and potential engagement of target mechanisms of a peer navigation intervention for justice-involved individuals with SMI who are re-entering the community after jail release.

## Methods

### Development phase

#### Project advisory board

In collaboration with our local community mental health system (Genesee Health System; GHS), we will establish a project advisory board of 6–8 justice-involved individuals with SMI who have served as peer navigators or supervisors in other settings. We will also include individuals who have served as peer mentors for justice-involved individuals with SMI. Our advisory board members will provide periodic input throughout the project, including providing their pragmatic experiences to guide manual adaptation and study recruitment.

#### Manual development

We will draw content from the Chicago Health Disparities Center (CHDC) peer navigators’ training manual developed for homeless African Americans with SMI and adapt it for use by justice-involved individuals with SMI. The CHDC manual was designed to help clients engage with and benefit from primary care. This manual will be our starting document because it has a strong evidence base [9, 16] and was found to be effective in linking a marginalized SMI population to primary care services [16]. Adaptation of the CHDC manual will involve keeping the basic structures and principles of the peer navigation intervention and expanding the manual to include re-entry specific components described in Table 1 below.

**Table 1:** The MAPS intervention manual

	Current contents	Population specific additions
**Instrumental support** for treatment engagement and recovery	Principles in helping relationships Basic helper principles in your life Advocacy Engaging people through goal setting	Steps to peer navigation before anticipated date of release Assist with completion of important paperwork Link individuals with organizations that provide ID services Ensure access to transportation Housing, employment, etc. in the context of a criminal record
**Informational support** for tx engagement and recovery	Provide information about food pantries Inform clients about sliding scale and fee waivers	Information about treatment options for SMI Help clients identify appropriate providers Information on accessing mental health, medical, and substance use care
**Emotional support** for tx engagement and recovery	Reflective listening skills Strengths-based model	Unconditional acceptance and reassurance Adherence support (encouraging adherence) Going with clients to agencies to provide emotional and practical support
**Social norms** around treatment engagement and recovery	Setting boundaries Managing burnout The big picture	Sharing own experiences with SMI and justice involvement Working with the justice system Acting as role models

The manual will be adapted and expanded to include specific information related to working with justice-involved individuals with SMI and potential ways to effectively engage with them after re-entry (see Table 1). For example, content related to working with the justice system, how to assist with paperwork, the art of self-disclosure, meeting basic needs of clients (i.e., housing, food), identifying providers, and access to community services will be added.

#### Focus group discussions

We will conduct focus group discussions (FGDs) to aid in manual development and to provide feedback to help ensure that the MAPS peer navigation intervention meets the needs of our target population. Five FGDs of 6–8 people each (for 30–40 people total) will be conducted: 2 groups with experienced peer navigators and 3 groups with individuals in jail with SMI. The FGDs will explore perspectives of potential clients and peer navigators regarding the effectiveness, acceptability, and gender and cultural appropriateness of peer navigator intervention and the most important approaches for them to take with justice-involved individuals with SMI. FGDs with peer navigators will ask about: (1) the most essential peer navigation skills, (2) lessons they have learned in their careers to date, (3) local services for individuals re-entering the community, and (4) how to initiate and maintain a culturally competent peer navigation service for individuals re-entering the community. We will also interview 4 peer navigators working with prisons from other states to understand experiences across the country using the topic guide described above. With potential client groups, we will explore (1) how peer navigators can best gain their trust, (2) their greatest needs in overcoming barriers to (post-release) treatment engagement, (3) what would assist their treatment engagement and how peer navigators could help with that, and (4) feedback on the proposed MAPS intervention outline. To understand the challenges after re-entry, we will include participants who have had re-entry experiences. The peer navigators’ focus group discussions will focus on how peer navigators can best help participants overcome misconceptions, mental health stigma, and other barriers to service linkage.

We will conduct qualitative coding and analysis of FGD data. FGDs will be facilitated by a moderator and a note taker. FGDs will be audio-recorded. The audio files will be transcribed verbatim. With the assistance of the Co-investigators, the Principal Investigator will lead the qualitative analysis and interpretation. Major topics and sub-topics will be coded. Additional codes will be generated for topics that invariably arise and that may have significance to the project. A descriptive summary will be developed to represent key content of each FGD. To enhance rigor and facilitate the quality of data management, we will use NVIVO version 12 [18]. We will conduct member checking with selected study participants to validate the fit, credibility, and transferability of the results from the FGDs. We will use a framework analysis technique [19]. We will create the coding framework in the NVivo structure, and coders will chart data into the provided framework. We will refine and adapt our intervention for our target population based on the recommendations of focus group participants.

#### Open pilot trial

We will conduct an open pilot trial with 15 justice-involved individuals with SMI who meet the same inclusion criteria as participants in the randomized trial. Participants will complete all research procedures (see below) and receive MAPS, allowing us to gain experience with and assess the feasibility of the intervention, strategies for recruitment and retention of participants, recruitment and supervision of peer navigators, and participants’ compliance with the study protocol. Participants will be requested to complete an intervention-specific End-of-Treatment Questionnaire [20] assessing perceived helpfulness of the intervention and their comfort with the research processes and assessments. We will also conduct exit interviews with participants and peer navigators. In the exit interviews, we will collect information on what went well, what needs to be improved or added to the manual.

During the open trial, we will carefully evaluate and document our training methods, recruitment and retention procedures, and the fit and adaptability of the treatment manual. We will collect data on what went well and what needs to be improved. We will use our experiences with the recruitment process, intervention, participant tracing, follow-up assessments, data from training of peer navigators, the End-of-Treatment Questionnaire, and feedback from the exit interviews to further refine the manual.

### Pilot study phase

#### Study design and setting

The study will be conducted in Genesee County in Flint, Michigan. We partnered with Office of the Genesee County Sheriff and the Genesee Health System, a community mental health center in Genesee County. The study participants will be recruited from Genesee County Jail and from a network of peer navigators working with the Genesee Health System.

#### Sampling and recruitment

The open trial (*n* = 15) and randomized pilot study (*n* = 40) will use the same broad inclusion criteria. Participants will be (1) incarcerated in the Genesee County Jail, (2) aged 18 or above, (3) with lifetime DSM-5 diagnosis of SMI (including primary psychotic disorder [schizophrenia, schizoaffective disorder, or delusional disorder], bipolar disorder, and/or a major depressive disorder with psychotic features) as assessed by the Structured Clinical Interview for DSM-5 (SCID-5) [21], and (4) anticipating release in the following 2 months. We will exclude individuals who (1) expect to be sentenced to prison (i.e., expect to go directly to prison, not home, from the jail), (2) cannot provide name and contact information of at least two locator persons, and/or (3) do not have access to any telephone. Some individuals are intoxicated, high, manic, and/or experiencing active psychosis when arrested and brought to the jail. We will exclude individuals who are too impaired to provide informed consent (i.e., are unable to respond coherently to the screening and consent process). If someone reports being or appears to be intoxicated or high, screening and consent procedures will be postponed until later, and jail protocols for referral to care will be followed.

The study research assistant (RA) will consent and screen potential participants privately. The RA will explain all aspects of the study, including confidentiality and its limits, and address questions. If the participant agrees, s/he will sign an informed consent form and complete the baseline assessment. The RA will offer to read the consent forms aloud. The RA will emphasize that enrollment in the study is completely voluntary. Those who consent to participate will be provided with a copy of the study information sheet and an informed consent document. The peer navigators have no role in the study assessments.

#### The control condition

The control condition for this study will be Standard of Care (SOC). SOC consists of SOC + monitoring and emergency referral, as is required to fulfill ethical obligations to trial participants. To determine the naturalistic effects and costs of adding peer navigation intervention, participants in both conditions can receive any other treatment available to them. Participants who may be receiving other treatments will not be excluded. As part of our service utilization assessment, we will carefully characterize SOC for each condition.

#### The intervention

The peer navigation intervention is based on social support theory [22]. Peer navigators will help participants overcome the significant structural barriers to treatment engagement at community re-entry by providing instrumental (i.e., practical), informational, and emotional support for treatment engagement through activities such as helping with the referral navigation process, keeping track of appointments and paperwork, following up with clients to make sure they went to appointments, problem-solving challenges, providing information about free care, helping clients make informed decisions about care by providing patient education, and creating peer connections. In addition, sharing their own stories of treatment and recovery and acting as role models shifts social norms toward treatment engagement and recovery [16, 23].

Participants assigned to the intervention group will receive the MAPS peer navigation intervention for 6 months (1 month pre-release, 5 months post-release). Approximately a month before release, the study research assistant will introduce consenting eligible individuals to a peer navigator who will help them navigate through community mental health, medical care, or substance use services. All peer navigators will disclose their history of criminal justice involvement, SMI, and/or co-morbid substance use during the initial introduction. In the weeks before release, the peer navigators will meet their clients at private locations within the jail to conduct strengths and needs assessments, fill out insurance forms, provide information on steps required to establish their insurance eligibility and residence IDs, and help set up community treatment appointments as needed. Peer navigators will maintain regular contact with participants at least once a week in the jail until they are released. Information obtained as part of the pre-release visits will be used to plan the rest of the peer navigation process after re-entry.

**Fig. 1:**
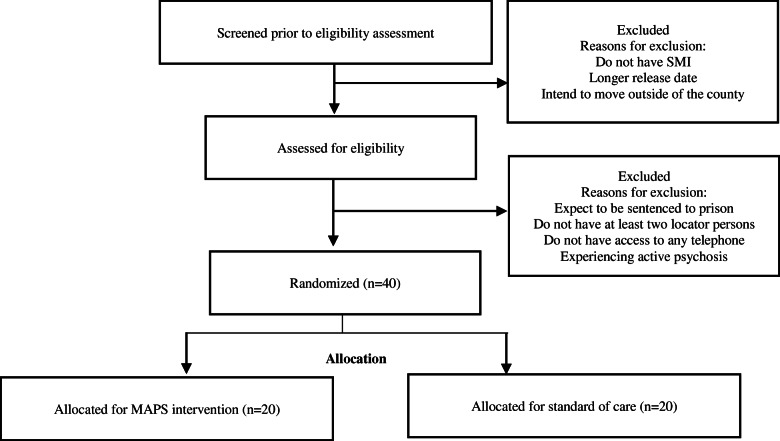
MAPS flow chart

After re-entry, peer navigators will meet with their clients (in person or by phone) up to three times a week in the first 2 months and once a week in the following 3 months after release for a session no less than 60 min. Peer navigators will meet their clients within the first week of re-entry, preferably within the first 24–72 h after release. In-person meetings will take place in the community at safe locations convenient for participants (i.e., libraries, treatment facilities, public buildings). The peer navigators will review participants’ activities since they left jail and problem-solve any barriers to service linkage or access that might have occurred. If clients have an outstanding referral to health care providers, the peer navigator will go with the client (whenever requested) or facilitate their trip to a provider by helping them get bus tickets or identify other reliable means of transportation. Peer navigators will meet with their clients depending on their need and jointly established treatment plan. Their services will involve (1) providing instrumental support for treatment engagement and recovery by focusing on strengths, being a role model and sharing personal experience, show genuine concern, identify stressors and roadblock and link to self-help [24]; (2) sharing information about treatment engagement, creating service linkages after involvement with the justice system, health and lifestyle changes required to promote recovery, and information about access to free treatment options [25, 26]; and (3) fostering emotional support for treatment engagement by showing empathy, promoting empowerment/hope, and facilitating trust in treatment providers [24, 27]. In addition to the weekly meetings with their clients, peer navigators will do a monthly progress evaluation and refine treatment plans accordingly. See the flow chart (Fig. 1) for the planned randomized trial below.

#### Peer navigator training and supervision

We will recruit 4 peer navigators (3 males, 1 female) and train them to fidelity to deliver the intervention. The 4 half-day long, didactic in-person initial trainings will consist of reviewing the peer navigation intervention rationale, materials, and strategies; audio-taped demonstrations; and live practice sessions with feedback. Weekly group supervision meetings will be held with peer navigators via phone or in-person to assist with any outstanding concerns. All peer navigation sessions will be audiotaped. Supervision will involve review of peer navigators’ audiotaped sessions (with fidelity rating as appropriate), group supervision and case discussion, and individual phone consultation as needed.

We will employ several approaches that we have found helpful in achieving low attrition rates (0–20%) in previous intervention studies with individuals re-entering the community after incarceration [28–31] (including those who were homeless) and other high-risk samples [32, 33]. These include study staff’s strong relationships with participants and efforts to value and appreciate their study participation. The study research assistant will call participants and mail them letters to remind them of their appointments and maintain a list of 2 other people who will always know where participants reside. Locator information will be updated at each study contact. Telephone follow-up assessment (removing the need for transportation) and study team flexibility in scheduling follow-up assessments (i.e., on evenings or weekends) will also facilitate participant retention. Finally, participants will be remunerated $50 for each follow-up assessment, facilitating retention.

#### Fidelity ratings

We will develop intervention adherence and competence ratings based on the intervention manual. Adherence rating scales will consist of checklists of tasks to be completed at each meeting. Competence rating scales will reflect peer navigator general skills, such as reflective listening skills, engaging people through goal setting, using a strength-based approach, and advocacy. We will use these scales to rate a randomly selected 33% of the open trial tapes. Scale development will continue until the item content is satisfactory, and interrater reliability is acceptable (> .80).

#### Assessments

##### Primary outcomes: feasibility and acceptability

The primary outcomes of this study will be to evaluate the *feasibility* and *acceptability* of the proposed recruitment methods and research design, of the intervention training methods, of delivering the enhanced peer-navigator and control interventions. We will use the End of Treatment Questionnaire [20] to assess client experience with the intervention. To measure the feasibility, acceptability and satisfaction with the intervention, we will use the Client Satisfaction Questionnaire (CSQ-8) [34]. We will also evaluate the recruitment, refusal, retention, attendance rates.

##### Secondary outcomes

**Clinical outcomes**

The presence and severity of manic symptoms will be measured by using the Altman Self-Rating Mania Scale (ASRM) [35]. This 5-item scale has been reported to be consistent with standard DSM 5 diagnosis of presence and severity of manic symptoms [35]. We will use the 16-item version of Prodromal Questionnaire (PQ-16) to assess psychotic symptoms [36] and the Quick Inventory of Depressive Symptoms (QIDS-SR) [37] to assess depressive symptoms. The 12-item WHO-Disability Assessment Schedule (WHODAS-II) [38] will be used to measure functioning in the domains of cognition, mobility, self-care, getting along/interaction with other people, life activities, and participation in community activities. The 4-item Brief Adherence Rating Scale (BARS) [39] will be used to assess adherence to psychiatric medications. BARS items evaluate past month adherence in terms of the number of prescribed doses per day, number of days the patient did not take the prescribed doses, and number of days the client took less than the prescribed doses. We will use Treatment History Interview (THI) [40] to measure number of hospitalizations after release. Numbers of suicide attempts will be measured by using the Columbia Suicide Severity Rating Scale (C-SSRS) [41]. Substance use will be measured using the Alcohol Use Disorders Identification Test (AUDIT) [42] and Drug Use Disorders Identification Test (DUDIT) [43] and brief measures of alcohol and drug use severity. The AUDIT has been recommended as part of the NIH PhenX Toolkit of assessments.

**Life context outcomes**

We will use a calendar-based interview method [44–50] to measure number of nights unstably housed and days until rearrest.

**Target mechanisms**

Measuring the target mechanisms for this peer navigation intervention may contribute toward refining the intervention, clarifying the peer navigator roles, and improving client outcomes. Instrumental support and informational support for treatment engagement will be measured using an adapted version of the Patient-Reported Outcomes Measurement Information System (PROMIS) measures, originally developed for people with chronic illness [51]. We will adapt the informational support (10 items) and instrumental support (5 items) sub-sections of the scale to assess the frequency and ease of obtaining these supports when needed. Emotional support for treatment engagement will be assessed using items from the Important People and Activities Scale [52]. The Self Stigma of Mental Illness Short Form (SSMI-SF) will be used to assess social norms around treatment engagement and recovery [53] (Table 2).

**Table 2:** Secondary outcomes of MAPS study

Health service outcomes	Utilization of community mental health (primary), medical, and substance use services; fewer days between release from jail and first contact with any health care provider.
Clinical outcomes	Reduced psychiatric symptoms, increased functioning, adherence to psychiatric medications, fewer substance using days, fewer hospitalizations, and suicide attempts.
Life context outcomes	Nights unstably housed and time until rearrest.
Potential target mechanisms	Instrumental (primary), informational, and emotional support for treatment engagement and social norms about treatment engagement and recovery.

##### Demographic/screening measures

Demographic/screening measures will include age, educational level, marital status, occupation, employment (status, number of hours per week), income, race, type of offense for which currently incarcerated, number of prior arrests, number of prior convictions, and length of sentence. These data will be compared to jail records. At follow-ups, occupation, employment (status, number of hours per week), and income will be repeated. We will use the Structured Clinical Interview for DSM-5 (SCID) [21] to ensure that all study participants have DSM-5 diagnosis of SMI, as operationalized in our inclusion criteria. The SCID is a semi-structured interview guide used for diagnosis of SMI. The 90-item Symptom Checklist 90 (SCL-90) [54] will be used to establish the eligibility criterion of “currently symptomatic” (using the SCL-90 score clinical cutoff of ≥25) [55]. Table 3 summarizes measurements and timepoints.

**Table 3:** Measurements and timepoints

Construct	Measure	Baseline/pre-release	3 months after release	6 months after release
Demographic and inclusion				
Demographics	Demographics	X		
Inclusion	SCID [21] and SCL-90 [54]	X		
Feasibility and acceptability				
Feasibility and acceptability	CSQ-8; ETQ; WAI			X
Health service outcomes				
Mental health tx utilization	THI	X	X	X
Substance use tx utilization		X	X	X
Medical tx utilization		X	X	X
Days to first contact with a provider			X	X
Clinical outcomes				
Mental health symptoms	ASRM [35]; PQ-16 [36]; QIDS	X	X	X
Functioning	WHODAS-12 [38]	X	X	X
Psychiatric medication adherence	BARS [39]	X	X	X
Psychiatric hospitalizations	THI	X	X	X
Suicide attempts	C-SSRS [41]	X	X	X
Substance use	AUDIT/DUDIT	X	X	X
Nights unstably housed, time to rearrest	Calendar-based interview	X	X	X
Target mechanisms				
Informational support for treatment	PROMIS [51]	X	X	X
Instrumental support for treatment	PROMIS [51]	X	X	X
Emotional support for treatment	IPA [52]	X	X	X
Social norms re: treatment and recovery	SSMI-SF [53]	X	X	X

#### Data analysis

The purpose of the R34 Exploratory Research Award is to collect preliminary data to assess feasibility and acceptability of an intervention and to inform a subsequent fully powered randomized clinical trial. With data from 40 participants in intent to treat analyses (about 20 per condition), we would only have statistical power adequate (.80) to detect large effects (*d* = .91) with alpha of .05. Randomized trials of SMI interventions rarely produce effect sizes this large; therefore, our primary emphasis will be on examining the direction of effects and the range of effect sizes for differences between conditions. This pilot data can be used to demonstrate whether the effects of treatment look promising across a set of outcome variables, to begin to examine distribution of outcome variables to inform future analytic strategies.

Primary analyses will be intent-to-treat (using data from all treatment enrollees). We will also conduct secondary dose-response analyses. Analysis strategies used (mixed linear models) can accommodate missing data and can be used in a sample of 40 with a simple model. We will use the STATA quantitative data analysis software [56].

##### Study feasibility and treatment feasibility/acceptability

We will assess the feasibility of the research procedures by examining study recruitment and refusal rates, participants’ willingness to be randomized, follow-up rates, reliability and range of responses to study questionnaires, and success of the peer navigator training program. We will assess the feasibility and acceptability of peer navigation program by examining rates of treatment attendance, rates of treatment completion (based on the jointly established treatment plan) and drop-out, and scores on the End of Treatment Questionnaire. We will also examine reasons for termination for consistent patterns. We will examine the acceptability of peer navigation intervention by using data from CSQ-8 treatment satisfaction questionnaire and detailed exit interviews. Peers’ experiences with the peer navigators, the quality of their working relationship, and their level of satisfaction with the service will evaluate using the Working Alliance Inventory-Short Revised (WAI-SR).

**Secondary outcomes**

We will (1) calculate the effect size and 95% CI for number of outpatient mental health appointments. Exploratory tests for differences between conditions will use mixed linear models, with number of outpatient mental health appointments in the 90 days prior to incarceration and baseline committed partnership status as a covariate. (2) Calculate the effect size and 95% CI separately for number of outpatient substance use appointments and number of outpatient medical appointments. (3) Conduct an exploratory comparison of the number of days between jail release and the first outpatient mental health appointment using Cox regression.

We will separately calculate the effect size and 95% CI for secondary outcomes, including mental health symptoms (ASRM, PQ-16, and QIDS-SR scores), functioning (WHODAS-12 score), psychiatric medication adherence (BARS score), number of post-release hospitalizations (from the THI), substance use (AUDIT and DUDIT scores), and nights unstably housed. Separate exploratory tests for differences between conditions will use mixed linear models, with baseline scores as covariates. We will conduct an exploratory comparison of time until rearrest using Cox regression.

**Target mechanisms**

We will separately calculate the effect size and 95% CI for the effect of the intervention on proposed target mechanisms including instrumental (primary), informational, and emotional support for treatment (measured using the PROMIS measures and IPA respectively) and social norms supporting treatment engagement and recovery (measured using the SSMI-SF). Separate exploratory tests for differences between conditions will use mixed linear models, with baseline scores as covariates. We will then explore the association of each of these target mechanisms with changes in our primary outcome (number of outpatient mental health visits) from baseline through 6 months post-release. These exploratory analyses will inform full tests of mediation (i.e., tests of the hypothesis that the effect of MAPS on outpatient mental health appointments is mediated through instrumental, information, and emotional support for treatment and social norms supporting treatment engagement/recovery) in a subsequent fully powered trial. Although we expect the MAPS intervention to be effective, should the intervention show no or limited evidence of effectiveness, exploratory tests of target mechanisms will also provide some initial information about whether MAPS’s limited effectiveness was due to failure to engage target mechanisms or to the target mechanisms not being associated with the final outcome.

**Personalization and processes**

We will explore gender, race/ethnicity, barriers to access to mental health care (assessed using the Barriers to Access to Care Evaluation Scale-Expanded; BACE-E [57]), substance use severity, number of past-year emergency room visits, and number of lifetime arrests as predictors and moderators of treatment outcome. We expect that MAPS will be appropriate for a full range of justice-involved individuals with SMI. We will also conduct preliminary analyses of dose-response effects.

**Treatment integrity**

We will compute scale reliabilities of adherence and competence ratings using both individual item correlations and total intraclass correlations. We will calculate scale validity by correlating adherence and competence ratings to intervention outcomes, to each other, and to expert ratings. Adherence and competence ratings of trained raters will be compared to expert global ratings to determine cut-off scores with sufficient sensitivity and specificity.

#### Ethics and dissemination

The Mentoring And Peer Support (MAPS) study was approved by Michigan State University Biomedical IRB (#17–772). All study staff also completed the Michigan State University Human Subject training certificate and good clinical practice (GCP) trainings. The study is also registered in www.clinicaltrials.gov under identifier #NCT04256954, date of registration 05 February 2020, https://clinicaltrials.gov/ct2/show/NCT04256954?term=NCT04256954&draw=2&rank=1.

The study will take several steps to protect the confidentiality of research data. The assessment survey and electronic data are linked using a subject ID number that is assigned upon enrollment into the project. Baseline data will be collected using standardized paper forms and will only be identified with the study ID of the participant. The codes that link the name of the participant and the study ID will be kept confidential in secured cabinets at the university. Collected forms will be transported to the PI’s data entry center at the university. Data collected from participants will be coded using that number rather than a name. Peer navigation sessions will be recorded using encrypted, password-enabled credit-card sized digital audio recorders that can also be brought to jail. Peer navigators then will upload the recordings to our secure research audio server from their (remote) computers for study supervision.

Results of this study will be shared with our local partners, including the Genesee County Jail, the Genesee Health System, and the Flint community. We will work with Office of the Genesee County Sheriff and our other community partners to share results of the study with their state and national networks, through presentation at their conferences and meetings, newsletters, flyers, and other strategies that they deem to be appropriate. We will also work with the university media representatives to disseminate results.

## Discussion

The current study is a formative work for a larger randomized clinical trial evaluating the effectiveness of peer navigation intervention for justice-involved individuals with SMI. The intervention aims to improve linkages with mental health, medical care, and substance use services during the vulnerable stage of community re-entry.

A growing body of evidence suggests peer provided services can potentially improve health outcomes of vulnerable populations [16, 58, 59]. Justice-involved individuals with SMI reentering the community after jail incarceration experience significant challenges that often limit their access to treatment [60, 61]. The social support provided by peer navigators in the form of informational support, instrumental support, and emotional support has the potential to improve linkages to services. Beyond these, peer-provided services also have the potential to improve clinical outcomes including reducing psychiatric symptoms, increase functioning, improve adherence to mental health medications, fewer substance use days, and fewer hospitalizations and suicide attempts [16]. The study is unique because our peer navigators are peers with their clients both in terms of justice involvement and history of SMI.

The study is innovative in many ways. First, this is the first randomized trial of peer navigation services for individuals with SMI re-entering the community after incarceration. Peers serve as crucial agents in identifying gaps in mental, physical, and substance use services for this group which is shown to experience multidimensional vulnerability [60, 62]. Second, we will involve individuals who are peers in a dual sense (i.e., having history of SMI and justice involvement). Peer navigation intervention may provide a potentially effective and cost-effective model for early initiation and maintenance of service linkage at community re-entry for individuals with SMI leaving jails. Finally, only a few RCTs of any intervention for re-entering justice-involved individuals with SMI focus on service linkage outcomes, which are arguably more proximal to clinical symptom outcomes. Most RCTs in this population focus on housing, family relations, etc. [63–65]. For example, critical time intervention (CTI) is another integrated case management model designed to reduce homelessness for individuals with SMI during vulnerable transitions (e.g., in and out of shelters or hospitals). Most of the 4–5 RCTs testing CTI for individuals leaving incarceration focused on homelessness [66], recidivism [67], and symptom severity with limited evidence regarding its effectiveness on post-incarceration service linkage [68].

Therefore, additional information about improving service engagement after release from jail is a contribution to the literature. This is the first study of reentering individuals with SMI to evaluate target mechanisms. By exploring MAPS’s engagement of target mechanisms and the relationship between the mechanisms and clinical outcomes, this trial will contribute to wider knowledge identifying and demonstrating how to engage therapeutic targets for our target population.

## Data Availability

N/A
